# An Evolutionary Microcircuit Approach to the Neural Basis of High Dimensional Sensory Processing in Olfaction

**DOI:** 10.3389/fncel.2021.658480

**Published:** 2021-04-30

**Authors:** Gordon M. Shepherd, Timothy B. Rowe, Charles A. Greer

**Affiliations:** ^1^Department of Neuroscience, Yale School of Medicine, New Haven, CT, United States; ^2^Department of Geological Sciences, Jackson School of Geosciences, University of Texas at Austin, Austin, TX, United States

**Keywords:** neuronal microcircuits, evolution, microcircuit, lateral inhibition, sensory dimensionality, olfactory processing, content addressable memory, mammalian paleontology

## Abstract

Odor stimuli consist of thousands of possible molecules, each molecule with many different properties, each property a dimension of the stimulus. Processing these high dimensional stimuli would appear to require many stages in the brain to reach odor perception, yet, in mammals, after the sensory receptors this is accomplished through only two regions, the olfactory bulb and olfactory cortex. We take a first step toward a fundamental understanding by identifying the sequence of local operations carried out by microcircuits in the pathway. Parallel research provided strong evidence that processed odor information is spatial representations of odor molecules that constitute odor images in the olfactory bulb and odor objects in olfactory cortex. Paleontology provides a unique advantage with evolutionary insights providing evidence that the basic architecture of the olfactory pathway almost from the start ∼330 million years ago (mya) has included an overwhelming input from olfactory sensory neurons combined with a large olfactory bulb and olfactory cortex to process that input, driven by olfactory receptor gene duplications. We identify a sequence of over 20 microcircuits that are involved, and expand on results of research on several microcircuits that give the best insights thus far into the nature of the high dimensional processing.

## Introduction

A brain microcircuit has been defined as the functional organization of nerve cells in specific patterns that carry out specific information processing tasks characteristic of a given brain region. This has provided an approach to analyzing different brain regions (Shepherd, 2004), including the evolution of the cerebral cortex from three-layer dorsal cortex present in amniotes ancestrally to six-layer mammalian neocortex (Shepherd, 2011). Here we apply this approach to the neural basis of sensory perception in the olfactory pathway, with the aim of identifying the microcircuits that enable the olfactory pathway to carry out processing of its high dimensional sensory input mostly within only two core regions, the olfactory bulb and olfactory (piriform) cortex, together with recent evidence regarding the closely related olfactory tubercle. The vomeronasal organ and terminal nerve are also components of the mammalian olfactory system (reviewed in Rowe et al., 2005) but our focus here is restricted to the main olfactory system that mediates conscious odor perception.

Olfactory input consists of a formidable array of thousands of different olfactory molecules (odorants) whose different unique chemical structures constitute a high number of “dimensions.” A fundamental question is how this high multidimensional array is reduced to representation in the two-dimensional sheets of the olfactory bulb and olfactory cortex to serve as the basis for olfactory perception.

We have previously introduced an evolutionary perspective on olfactory processing (Rowe and Shepherd, 2016) and use that again here because olfaction provides an excellent example of Dobzhansky’s tenet that nothing in biology makes sense except in the light of evolution. While our focus is on the elaborate neural microcircuits in extant Mammalia (Rowe, 2020b), paleontology adds unique insights into the evolution of this complex system because the vertebrate brain and many of its peripheral sensory systems require a rigid framework within which to function properly (Rowe, 1996a, b). The olfactory system is no exception. There is a rich and well-studied fossil record of ‘stem-mammals’ – i.e., the extinct relatives of the crown clade Mammalia – and their skulls preserve evidence of evolutionary transformations in the olfactory bulb, olfactory cortex, and bones that develop from the embryonic olfactory capsule to support an olfactory epithelium that eventually evolved into the most expansive olfactory receptor array of any vertebrate. Additional evolutionary insights are provided by using the ‘extant phylogenetic bracket’ (Witmer, 1995) in which living mammals and reptiles are compared in order to draw inferences about properties of the olfactory genome, soft tissues, and ontogenetic mechanisms in amniotes ancestrally. These mark the starting point from which the mammalian lineage – “Synapsida” or in phylogenetic terminology “Pan-Mammalia” (Rowe, 2020e)– diverged from the ancestral amniote onto its own unique evolutionary trajectory.

It will come as no surprise that the remarkable complexity of olfactory microcircuits described here for living mammals emerged over hundreds of millions of years, as stem-mammals found compounding success through increased focus on solving the problem of odorant high dimensionality in the rapidly changing chemical environments they faced (Rowe et al., 2011; Yohe et al., 2020). Duplications in olfactory receptor genes played a special role in events leading up to the origin of Mammalia and neocortex (Rowe and Shepherd, 2016; Rowe, 2020f). This was before the other sensory systems began to evolve significant processing at the cortical level. It is exciting to recognize that evolving olfactory microcircuits thus led the way in cortical processing of high dimensional input among sensory systems.

Here we provide a summary of classical and recent studies that show more than 20 intricate and tightly interconnected local neuronal microcircuits for information processing within the olfactory pathway. As shown in Table 1, these microcircuits exist at different levels of organization, from molecular, to integrative units within individual neurons, to neurons, neuron microcircuits within a region, and microcircuits between regions. Given the high dimensionality of the odor molecule stimulus, the aim of this review is to frame the hypothesis that these olfactory microcircuits are highly integrated in sequence and in parallel for dimension reduction. This obviously is only a start to pointing the field toward filling in the gaps for a comprehensive understanding of the neural basis of odor perception.

**TABLE 1:** Enhanced sensory processing microcircuits in the mammalian olfactory pathway.

Enhanced processing units	Olfaction
Multiple molecular structures	Odor stimuli
Multiple molecular interactions	OSN transduction
Multiple spatial representations (maps)	Olfactory bulb glomeruli
Multiple integrative units in neurons	Granule cell spines, mitral cell dendrites
	Olf Cortex Pyramidal cell dendrites, spines
Multiple function neurons	Granule cell lateral inhib and oscillation
Multiple function microcircuits	Granule cell lateral inhib and oscillation
Multiple microcircuits within a region	Olfactory bulb lamination
Interregional microcircuits	Olfactory bulb plus olfactory cortex

*Key: *Olfaction: OSN, olfactory sensory neuron; Olf, olfactory; inhib, inhibition.**

## Microcircuit Organization of the Olfactory Bulb

**FIGURE 1:**
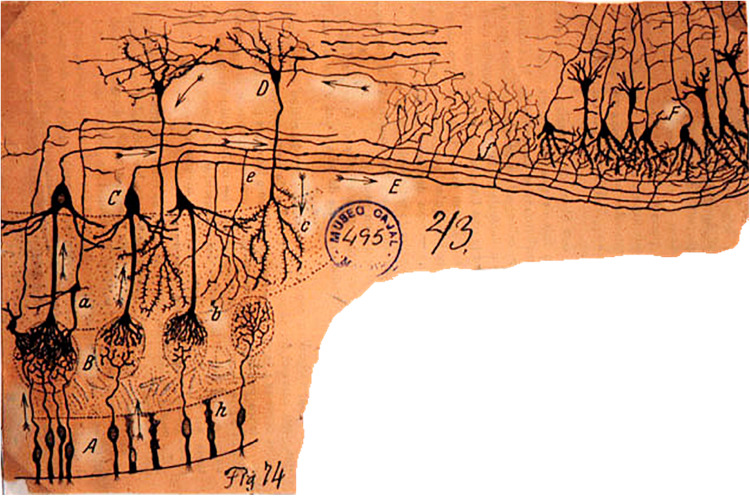
The cellular composition and basic connectivity of the mammalian olfactory pathway according to Ramon y Cajal. A, olfactory sensory neuron; B, olfactory axon endings in olfactory glomerulus; C, mitral cell; D, granule cell; E, lateral olfactory tract; a, tufted cell; b, terminals of mitral cell recurrent axon branch; c, granule cell dendritic branches; e, mitral cell recurrent axon. On the right, the beginning of the anterior olfactory cortex and piriform cortex. Ramon y Cajal, Cajal Institute. From Cajal (1894); see also Figueres-Oñate et al. (2014).

When Camillo Golgi invented the Golgi stain to visualize single neurons, his first published pictures were of the cells of the olfactory bulb (Golgi, 1875). Similarly, when Santiago Ramon y Cajal carried out his first Golgi studies, the olfactory bulb neurons were among the first he reported (Cajal, 1890). As shown in Figure 1, they consist of the mitral cell (so-called because its cell body has the shape of a bishop’s miter), which sends a single dendrite to ramify and interconnect with the ramifications of olfactory nerve fibers within a spherical region called a glomerulus. Between glomeruli are cell bodies of small neurons called variously periglomerular (PG), juxtaglomerular, and external tufted cells. The mitral cell also gives rise to several lateral dendrites which extend and branch sideways to ramify among dendrites of a cell so small it was called a granule cell, to form with them the external plexiform layer. The granule cell lacks an axon, so for many years it wasn’t known whether it was in fact a nerve cell. The external plexiform layer also contains recurrent branches of the axons of mitral cells, and a smaller version of mitral cells called tufted cells. The granule cell bodies are in a deep layer along with other cells with short axons.

The foundation for the present review began with one of the first attempts to analyze the functional organization of the olfactory bulb based on the Golgi anatomy and electrophysiological data (Phillips et al., l963). This produced one of the first brain microcircuit diagrams, in which olfactory input into an olfactory glomerulus activates the mitral, tufted and periglomerular cells connected to it, which is followed by a long lasting lateral inhibition of the mitral and tufted cells ascribed to the granule cells (Shepherd, 1963a, b). A subsequent study provided the first compartmental computer model of dendrodendritic interactions between mitral and granule cells as the basis for the lateral inhibition (Rall et al., 1966; Rall and Shepherd, 1968).

This was followed by a study of the functional organization of the olfactory cortex, which provided a summary view of both feedback and lateral inhibition and feedback and lateral excitation (Haberly and Shepherd, 1973). It has been suggested that this organization of three-layer cortex is the ancestral state from which the organization of six-layer forebrain cortex evolved, a concept supported by Kriegstein and Connors (1986) and by Rowe and Shepherd (2016). This was followed by path breaking studies of Haberly and Bower (1984); Haberly (1985), and Haberly and Bower (1989) (see also Doucette et al., 2011; Martin-Lopez et al., 2019) with evidence that the olfactory cortex is not a simple relay from the olfactory bulb to the neocortex, but rather functions as a higher associative cortex analogous to the face area in the visual neocortex.

Any overview of olfactory microcircuits has the advantage of the breakthrough of Buck and Axel (1991) in identifying the olfactory receptor genes and olfactory receptors (ORs). Our own work at this level builds on the first computational models of odor molecule-receptor binding (Araneda et al., 2000; Singer, 2000), the pharmacology (Shepherd and Firestein, 1991) and electrophysiology (Zhao et al., 1998) of the olfactory sensory neuron (OSN) odor response, and the routing of the axons from OSNs expressing the same OR to the same olfactory glomerulus (Singer et al., 1995; Mombaerts et al., 1996). The complexity of mammalian olfactory organization can be appreciated from studies in the mouse, in which odorant discrimination relies on nearly 1,000–1,200 different OR types which can be grouped into subfamilies that recognize different structural classes of odorant molecules (Ressler et al., 1993; Touhara et al., 1999; Ma and Shepherd, 2000; Zhang and Firestein, 2002; Godfrey et al., 2004).

## Evolutionary and Developmental Perspective on Organization of the Olfactory Bulb

Based on comparisons among living species it has been estimated that the ancestral mammal had ∼1,200 OR genes, whereas the ancestral amniote and ancestral vertebrate had only ∼100 olfactory genes (Rouquier et al., 2000; Niimura and Nei, 2006; Niimura, 2012; Niimura et al., 2014; Zhou et al., 2021). OR gene duplications occurring over ∼160 million years of stem-mammal evolution must have played a central role in the emergence of the complex microcircuit network inferred to have been present in the last common ancestor of Mammalia. The impact of these gene duplications can be more fully appreciated in a cascade of ontogenetic interdependencies that follow OR gene expression. The number of expressed OR genes has been found to correspond to olfactory epithelium surface area, ethmoid turbinal surface area, total area of foramina in the cribriform plate, olfactory bulb size, and olfactory cortex size (Pihlström et al., 2005; Rowe et al., 2005; Hayden et al., 2010; Schlosser, 2010; Bird et al., 2014, 2018; Garrett and Steiper, 2014; Rowe and Shepherd, 2016).

The ORs are expressed in large populations of OSNs. In an early study of the rabbit, Allison (1953) estimated an average of 120,000 OSNs per mm^2^ of olfactory epithelium based on optical microscopy and histology. We have used computed tomography to measure the surface areas of turbinals supporting the olfactory epithelium in a variety of mammals (e.g., Rowe et al., 2005). Extrapolating Allison’s estimate to different species, we can project that the opossum *Monodelphis* has ∼145 million OSNs in its nose, and the long-beaked echidna has ∼1.3 billion OSNs. Even with smaller estimates (up to 100 million in dogs; 10–20 million in rodents, 10 million in humans), the numbers nevertheless convey a sense of the immense computational problem that mammalian olfaction poses. That the number of OSNs can vary by roughly an order of magnitude between different mammalian clades is an indication of olfaction’s evolutionary impact on mammalian diversification.

The size of olfactory bulb and cortex has important morphogenic consequences for organization of the skull in mammals that can be traced back into the deep fossil record of stem-mammals. These individual bony components of the olfactory system do not exist as separate parts independent of the rest of the system; they develop in concert with one another in mammalian ontogeny (Rowe et al., 2005). Each offers a proxy for the system as a whole, and thus the fossil record, fragmentary though it is, can help identify correlations and sequences of evolutionary transformations across the olfactory system, and the approximate timings of these events. This approach describes the larger evolutionary and developmental context in which the microcircuitry detailed here evolved.

## Olfactory Epithelium and Its Skeleton

Olfaction is the first of the mammalian sensory systems to differentiate during development. Both the main olfactory epithelium and vomeronasal organ develop from a single pair of ectodermal olfactory placodes that form at the rostral extremity of the neural plate (Schlosser, 2010; Rowe and Shepherd, 2016). Soon after gastrulation, they invaginate to contact the rostral end of the neural tube, initiating organogenesis and the formation of their imminent synaptic connections to the presumptive olfactory bulb. The rostral position of the olfactory placodes may explain why olfaction is the only sensory system that projects directly to the telencephalon; the other cranial sensory placodes including the optic placode are positioned laterally or caudal to the presumptive diencephalon, consistent with why the mature visual pathway to the telencephalon is via the thalamus (Schlosser, 2010; Rowe and Shepherd, 2016).

The mature olfactory epithelium becomes arrayed over the surfaces of the nasal septum and, laterally and posteriorly, is supported by a skeleton of paper-thin filigreed scrolls, arbors, and plates of bone known collectively as turbinals (or turbinates). Mammals are unique in the extent to which this internal skeleton is elaborated (Gauthier et al., 1988; Rowe, 1988; Rowe et al., 2011). In the mouse and opossum turbinals support a ten-fold increase in the surface area of olfactory epithelium that can be contained within the volume of the nasal capsule (Rowe et al., 2005). The importance of this skeleton is amplified by understanding its early development (Rowe and Shepherd, 2016). Once initiated, growth of the olfactory epithelium over the inner walls of the olfactory capsule soon exceeds the capsule’s surface area, and folds of epithelium begin to grow into its lumen. This in turn induces a supporting skeleton of cartilage that grows apically into the folds. The overall effect is growth of rigid funnels whose diameters increase from tiny apertures at the cribriform plate into much wider mouths within the lumen of the olfactory recess of the nose. Perichondral ossification rapidly replaces the turbinal cartilages, starting from their bases, such that at no time in ontogeny is there a free-standing elaborate skeleton made only of cartilage (Rowe et al., 2005). From the first induction of the olfactory epithelium, its turbinal skeleton allows for unprecedented growth of epithelial surface area, while sustaining an unbroken tissue connection to the developing olfactory bulb. This developmental pathway was probably inherited from the ancestral amniote, but owing to the 10-fold increase in OR genes, it finds its most elaborate expression in mammals. The significance of this is discussed below in regard to growth and function of OSN axons.

The mature turbinals form a complex skeleton providing a rigid armature that maintains geometrically complex passageways and blind air-filled spaces known as “ethmoid cells” that are lined with olfactory epithelium, into which odorant molecules volatilize to reach ORs. Axons from OSNs (see below) expressing a particular OR gene converge on a single glomerulus, meaning that in the lateral aspect of the olfactory bulb each glomerulus receives impulses from a segregated and independent collection of OSNs, and that physical zones on the olfactory epithelium surface thus correspond to particular groups of glomeruli (Ressler et al., 1993, 1994; Mori et al., 1999). The turbinals and ethmoid cells therefore provide for sequestration of regions in the nose in which particular OR genes are expressed (Ressler et al., 1993; Rowe et al., 2005). Experimental evidence is lacking, but this sequestration suggests that odorant molecules with differing volatilities may be sorted in the nasal cavity in many mammals in an initial step that begins to reduce the multidimensionality of the odor molecules.

## Olfactory Sensory Neuron Cilia

Olfactory processing starts with the arrival of the odor molecules at the ORs located in the membranes of cilia that protrude from the dendritic knob of the OSN. The cilia themselves are bathed in an aqueous mucus layer through which the odor molecules are absorbed, which places its own physical limits on the access of the odor molecules as determined by their aqueous and lipid solubilities. This was recognized to be equivalent to a kind of chromatographic separation of odor molecules, hence the chromatographic theory of olfaction (Mozell, 1970). This theory can be adapted to incorporate the local volatile sequestration that occurs in the ethmoid “cells” or recesses described above. Here our focus is on the neural stages of processing. Discussion is expanded for those steps best understood; others are mentioned in hopes of stimulating further research.

### Interaction of Odor Molecules With Olfactory Receptors Occurs in the Olfactory Cilia

There is evidence of a rich field of pharmacological agonism and antagonism between the many different types of odor molecules and the many different types of ORs that greatly enlarge and elaborate the scope of activation of the receptors (Shepherd and Firestein, 1991; Xu et al., 2020).

### Mucus Actions

There is the likelihood of time dependent enzymatic actions on the odor molecules in the olfactory mucus (Getchell et al., 1984) which will add to, or reduce, the differentiation of responses of different ORs.

## Olfactory Epithelium Interactions?

Lateral interactions between OSNs have been reported in the antenna of *Drosophila*, within tightly structured olfactory cartridges, but not thus far in the more open structure of the mammalian olfactory sensory epithelium.

## Olfactory Sensory Neuron Axons

### OSN Projections to Olfactory Glomeruli Spatial Patterns

**FIGURE 2:**
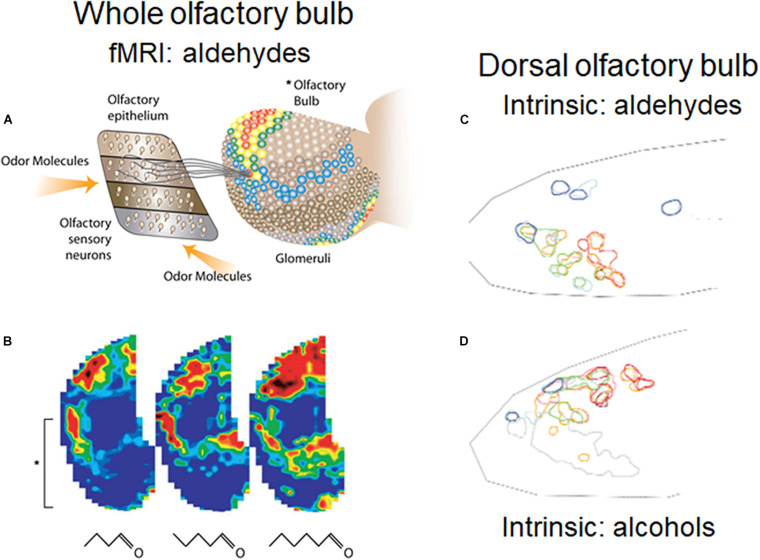
Orderly spatial activity patterns elicited in the rodent glomerular sheet by different odor molecular types using different methods. **(A)** Schematic view of olfactory epithelium divided into four zones, with representative OSNs projecting to a single glomerulus in the olfactory bulb (OB). In the OB, fMRI activity patterns are elicited in the entire glomerular sheet by a four carbon aldehyde. Red: strong response; yellow: moderate response; blue: weak response. **(B)** Maps of activity elicited in the whole OB by 4, 5, and 6 carbon aldehydes. Brackets show the part of the lateral OB seen in **(A)**. **(C)** Results of optical experiment limited to the rodent dorsal OB, recording intrinsic signals of responses to aldehydes of increasing carbon length, from short (red), medium (green), and long (blue) carbon chains. Map orientation of the dorsal OB is lateral up, medial down, anterior left, posterior right. **(D)** Intrinsic signals in the dorsal OB for stimulation with odors due to alcohols of increasing carbon lengths. The experiments confirmed burst firing of cells recorded in the circled areas. **(A,B)** Xu et al. (2003); **(C,D)** Uchida et al. (2000).

A general rule in mammals exemplified by rodents is that axons of OSNs expressing a given OR project to the same glomerulus on either the medial or lateral aspect of the OB (Ressler et al., 1994). The distribution of the glomeruli representing different ORs means that there is a spatial organization to the glomeruli, and to the olfactory bulb neurons connected to them. The different glomeruli are differently activated by their different OR inputs, producing spatial patterns of activated glomeruli, as first shown with 2-deoxyglucose in the awake behaving rat (Sharp et al., 1975) and widely documented by many methods in many species (summarized in Uchida et al., 2000; Xu et al., 2000; Johnson and Leon, 2007; Mori and Sakano, 2011; Sanganahalli et al., 2020). An example of patterns generated by a homologous series of aldehydes recorded by fMRI is shown in Figures 2A,B, and by aldehydes and alcohols recorded by intrinsic imaging in the dorsal olfactory bulb in Figures 2C,D. The recordings with the different methods likely represent more widespread activation patterns through much of the glomerular sheet (Bisulco and Slotnick, 2003; Johnson et al., 2009). The extensiveness of the patterns and the redundancy built into them makes them resistant to damage by noxious and infectious actions on the OSNs (Bisulco and Slotnick, 2003; Johnson et al., 2009). Given the dependence of most mammals on olfaction, this capacity has been crucial to survival. Evidence is presented that a number of the microcircuits reviewed here process this spatial information at successive levels of abstraction at successive stages in the olfactory pathway.

### Ephaptic Interactions

The OSN axons emerge beneath the basal lamina of the olfactory epithelium and begin to bundle with other axons within specialized glial cells, olfactory ensheathing cells (OECs), that are coupled via gap junctions (Rela et al., 2010). These are among the thinnest axons in the nervous system – 0.2 μm in diameter – similar to parallel fibers in the cerebellum. Their action potentials have conduction rates of 0.5–1 mm/sec. One OEC may enclose from ten to several hundred axons, meaning a lot of membrane-to-membrane appositions, which raises the possibility of local currents generated by an action potential in one axon influencing the excitability of neighboring axons, referred to as an ephapse. As noted this has been reported for interactions between olfactory cells in *Drosophila*, but the evidence has been equivocal in mammals. These interactions can take the form of lateral excitation, involving excitatory ephaptic effects of local currents, or lateral inhibition due, for example, to accumulation of K+ in the extracellular space, including effects of K+ extruded during action potential activity.

During development this grouping (fasciculation) of axons begins before the cells express their OR gene (Miller et al., 2010; Liberia et al., 2019). Fasciculation is facilitated by the funnel-shaped geometry of the turbinals. Olfactory epithelium is present in the mouths of the funnels, segregating separate ethmoid cells, and as their OSN axons grow they are funneled by their bony enclosure toward particular tiny openings in the cribriform plate that direct axons to enter the olfactory fossa and to arrive on the olfactory bulb surface in localized areas (Rowe et al., 2005). OR gene expression in OSNs begins a day or two before a fascicle of axons enters a specific glomerulus, whereas the common “olfactory marker” gene in their cell bodies begins expression after innervation (Rodriguez-Gil et al., 2015; Liberia et al., 2019).

Olfactory sensory neurons are short-lived and new cells are generated throughout ontogeny from stem cells in the base of the olfactory epithelium. With the evolution of a ten-fold increase in OR genes, OSNs, and glomeruli the problem of axonal guidance was amplified proportionately, and how the axons of these new OSNs find their paths across broad expanses of epithelium to reach their target glomerulus has been difficult to explain (Mombaerts, 1999; Dryer, 2000; Mori and Sakano, 2011; Liberia et al., 2019). As noted, 3D imaging of mammals indicates that the shape of each turbinal resembles a funnel, which grows from the tiny foramina of the cribriform plate rostrally toward its mature, wide-mouthed terminus (Rowe et al., 2005). Early developmental studies (Harrison, 1910) showed that axonal guidance was determined by the geometry of their physical substrate; they grow in a straight line along solid substrates or follow topographic paths when such paths are visible (Harrison, 1914). The funnel-shape of each turbinal promotes fasciculation and passively directs growing axons toward the cribriform plate.

Interactions between OSNs expressing the same OR have been suggested to play a role in recognition between axons converging on their mutual target glomerulus (Singer et al., 1995). Across most of stem-mammal evolution, ephaptic interactions with existing axons from identical OSNs must have been a prominent mechanism in axonal guidance. The elaborate turbinal skeleton was a relatively late development in stem-mammal evolution that emerged as expansion of the olfactory bulb and olfactory cortex approached mammalian proportions (Rowe et al., 2011).

## Olfactory Bulb

**FIGURE 3:**
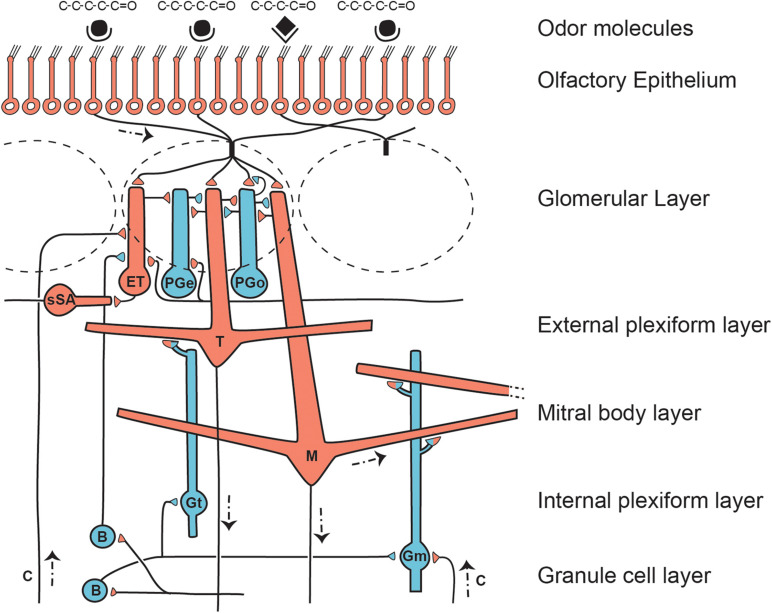
Microcircuit organization of a glomerular unit. B, Blanes (deep short- axon) cell; c, centrifugal axon; ET, external tufted cell; Gm, mitral connected granule cell; Gt, tufted cell connected granule cell; M, mitral cell; PGe, periglomerular cell (input from ET cell); PGo, periglomerular cell (input from OSN); sSA, superficial short-axon cell; T, middle tufted cell. Excitatory actions shown by red cells and terminals; inhibitory by blue cells and terminals. All cells except the M and T cells turn over during life. Note the multiple layers for lateral inhibitory and related processing actions. The complex patterns of glomerular synapses are under active investigation. From Shepherd et al. (2020), representing a synthesis of studies by multiple authors, including Cleland and Sethupathy (2006); Wachowiak and Shipley (2006), Migliore et al. (2010); Nagayama et al. (2014), Burton and Urban (2015), and Cavarretta et al. (2016).

As indicated in Figure 3, there are multiple distinct types of lateral interactions within the olfactory bulb layers.

## Glomerular Level

### Glomeruli

As noted by the classical histologists (Figure 1), the most distinctive anatomical feature of the olfactory bulb is the rounded islands of neuropil called glomeruli, containing the endings of OSN axons which synaptically transfer their signals to the dendritic tufts of mitral, tufted, and glomerular layer cells. Glomeruli thus place strict lateral spatial constraints on the projections of the OSN axons and the dendrites of the bulbar cells that only with the identification of OR genes have begun to be understood. The most salient fact is that in most cases all of the input to a given ordinary glomerulus is from axons from OSNs that express the same OR gene (Mombaerts et al., 1996). Of the approximately 3,500 glomeruli found in the mouse olfactory bulb, 2/3 are targeted by each of the ∼1,200 ORs yielding an overall convergence ratio of ∼3:1 (Richard et al., 2010). There is also a correlation in mice between the number of OSNs expressing an OR and the volume of the targeted glomeruli. For example, the OR P2 is expressed in ∼15,000 OSN while the OR M71 is expressed in ∼2,500 OSNs. The volumes of their target glomeruli are ∼1,500,000 μm^2^ and ∼500,000, respectively (Bressel et al., 2016). The relationship between the number of intact ORs in humans and their target glomeruli is less clear. Human have ∼400 intact ORs but there are >5,500 glomeruli yielding a convergence ratio of ∼16:1 (Maresh et al., 2008).

A corollary is that each glomerulus receives signals from an area or zone in the olfactory epithelium. In effect, this reduces the problem of the overall high dimension of the entire olfactory world to only one or a few dimensions within each glomerulus. This suggests that the microcircuits within each glomerulus process that part of the odor world, and microcircuits between glomeruli begin the process of knitting the separate odor mini-worlds together.

Insight into dimension reduction in the glomerulus is provided by the honeybee antennal lobe, equivalent to the mammalian olfactory bulb. Glomeruli in the honeybee are divided into a medial and lateral group. Using calcium imaging of the projection neuron responses to odor stimuli, Carcaud et al. (2018) found that medial glomeruli were more sensitive to an odor molecule’s functional group (alcohol, ketone, and aldehyde) whereas lateral glomeruli were more sensitive to chain length (C6 to C9). Evidence was cited that this differentiation of quality was due to a network of heterogeneous local interneurons (using glutamate, histamine, or various neuropeptides as neurotransmitters) that bring about contrast enhancement of differences in odor quality through lateral inhibition within the glomeruli.

It was concluded that the “two subsystems provide higher order brain centers with different, but complementary portions of odor quality information” (Carcaud et al., 2018). Thus far there is no evidence in mammals of this differentiation of ordinary canonical glomerular microcircuits related to odor molecule structure and function. Exceptions are glomeruli that receive a special input. These include the modified glomerular complex related to suckling (Greer and Shepherd, 1982), necklace glomeruli based on cyclic G sensory transduction (Zufall and Munger, 2010), and the trace amine-associated receptors (TAARs) (Liberles and Buck, 2006). For the ordinary glomeruli, one can hypothesize that the explosion of OR genes in early mammalian evolution, combined with marked dietary diversification in mammals and their close extinct relatives (see below), meant that the olfactory glomerular microcircuits became mainly adapted to process multiple chemical types and odor qualities across a far wider odor spectrum. This differential processing could be a function of the network of interneurons at the glomerular level (see Figure 3). The network could heighten the discrimination of odor quality dependent on learning mechanisms activated during the history of odor exposure and the behavior elicited (Cavarretta et al., 2016). Evolutionary increases in numbers of glomeruli and expansion of the network can be inferred at several points in stem-mammal evolution where endocasts indicate relatively larger olfactory bulbs (below). We do not yet know the identity of these microcircuits, but we hypothesize that they are there.

Beyond these examples it is well to realize that a cluster of synaptic connections can be useful for various processing tasks, such as the barrel column for processing input from a whisker pad in a rodent and the cortical column in the visual cortex. These clusters may or may not be present in different species. Horton and Adams (2005) showed for example that the cortical column is an inconstant presence in visual cortex. Moreover, whiskers are not universally present in eutherian (placental) mammals (Catania and Catania, 2015); ancestral state reconstruction suggests that they evolved independently as many as seven times among eutherians (Muchlinski et al., 2020). In contrast, glomeruli are present in virtually all olfactory pathways from nematodes to humans, indicating that they provide a universal function for processing high-dimensional odors (Chen and Shepherd, 2005). Our focus is on this canonical pathway and how it mediates high dimension odor processing in mammals. An obvious general function of the convergence of similarly tuned OSNs is enhancing signal-to-noise (Chen and Shepherd, 2005).

### Olfactory Nerve Intraglomerular Connections

A key constraint on olfactory microcircuit organization is that an OSN axon does not branch on its way to the olfactory bulb (Land and Shepherd, 1974; Klenoff and Greer, 1998), and projects only to a single target glomerulus together with other axons from cells expressing that receptor. An OR typically has a graded sensitivity to a range of odor molecules, analogous to the graded sensitivity of color photoreceptors to different wavelengths of light. The interpretation of this organization is that this is a mechanism for organizing the processing of a wide variety of odor molecular conformations by giving each glomerulus an identity to a graded sensitivity within this variety. Ordinary OSN synapses are glutamatergic. The first microcircuit representation of this connection (Shepherd, 1963a, b) specifically left unresolved the question of monosynaptic or polysynaptic coupling between nerve terminals and intraglomerular dendrites in mammals. It is apparent now that the caution was justified; the synaptic relation may vary in different species. Gire et al. (2012) describe a multistep microcircuit involved in activation of mitral cell dendrites by olfactory nerve terminals in rodents. Each synaptic step gives an opportunity for spatial and temporal microcircuit processing.

### Gap Junctions

The first type of synaptic lateral interaction occurs at the point of initial response to olfactory nerve input within a glomerulus, by gap junctions between distal branches of tuft dendrites (Schoppa and Westbrook, 2001; Migliore et al., 2005). Any difference in potential between two interconnected dendritic branches will be equalized, promoting synchronization of the mitral, tufted and periglomerular (PG) cells belonging to the same glomerulus. Computationally, this implements a many-are-equal computation at the very onset of odor processing (e.g., Brody and Hopfield, 2003; Migliore et al., 2005). Subsequent processing within the glomerulus thus occurs on the functional foundation of membrane potential equalization and synchronization. This supports the idea that a glomerulus functions to reduce processing of its part of the odor world to one or a few dimensions.

An important feature of this effect revealed by biophysical modeling (Migliore et al., 2005) is that it occurs at an electrotonic distance from the processing that takes place through granule cell inhibition at the level of the mitral cell body (see step 12 below). The model shows how these two contrasting actions need to be electrotonically separated in order to achieve their different functions; simplification of a mitral cell to a single compartment, as in network models, loses this effect.

Gap junctions in general are more abundant in early development. One may hypothesize that this helps to establish the glomerulus as a functional unit, followed by excitatory and inhibitory chemical synapses developing to elaborate the processing that occurs in relation to the synchronous intraglomerular sensory input, distinct from further processing by networks at the glomerular input level, and at the output level by granule cell inhibition. They are also believed to contribute to the plasticity of glomerular microcircuits during early learning (Wu et al., 2020).

### Intraglomerular Inhibition: Dendrodendritic Inhibition

These are a subset of the heterogeneous intraglomerular interactions covered in Figure 3. Intraglomerular dendrites are interconnected by numerous dendrodendritic synapses. OSN terminals activate dendrites belonging to a subset of PG cells (see PGo in Figure 3) that through dendrodendritic synapses inhibit weak responses of tuft branches of mitral and tufted cells. This could generate an activation pattern resembling lateral inhibition in relation to more strongly activated glomeruli with similar molecular receptive range (Cleland and Sethupathy, 2006; Cavarretta et al., 2018). Activation is by glutamate, inhibition by GABA and by dopamine. This is only one of an increasing number of microcircuits that may be involved in processing within the glomerulus. Another example is control of on/off glomerular signaling by a local GABAergic microcircuit, giving the glomerular response an all-or-nothing quality (Gire and Schoppa, 2009), a mechanism suggested to be effective in odor coding (Koulakov et al., 2007).

### Feedback Inhibition Onto OSN Terminals

PG cell dendrites also feedback inhibition onto the OSN terminals, to modulate through GABA the amount of excitation by glutamate. Here as at other GABAergic synapses, in early development GABA may have a depolarizing effect due to postsynaptic transmembrane ion gradient differences.

### Interglomerular Lateral Inhibition

Another subset of periglomerular cells (PGe in Figure 3) is involved in interglomerular interactions through their axons that extend up to 6–8 glomeruli from their home glomerulus. The activation sequence is OSN terminals to dendrites of external tufted cells to superficial short axon cells, whose axons have excitatory synapses on distant inhibitory PGe cells (Aungst et al., 2003). This inhibition is believed to be complementary to the lateral inhibition mediated by granule cells onto the lateral dendrites of mitral and tufted cells (see below).

### Interglomerular Lateral Excitation

A subset of PGe cells connects to distant external tufted cells to keep the sequence going laterally within the glomerular layer. This network of connections thus has its own specific input from olfactory nerve terminals, and its own distinctive output targets for lateral inhibition and lateral excitation between glomeruli (Figure 3). The balance between excitation and inhibition is believed to be involved among other things in concentration encoding, perhaps by maintaining the balance between the two so that concentration is signaled by broader activation of glomeruli (Shao et al., 2019). Glutamate is the excitatory transmitter at these synapses and GABA the inhibitory transmitter.

## External Plexiform Level

### Mitral Cell Dendrodendritic Lateral Inhibition

**FIGURE 4:**
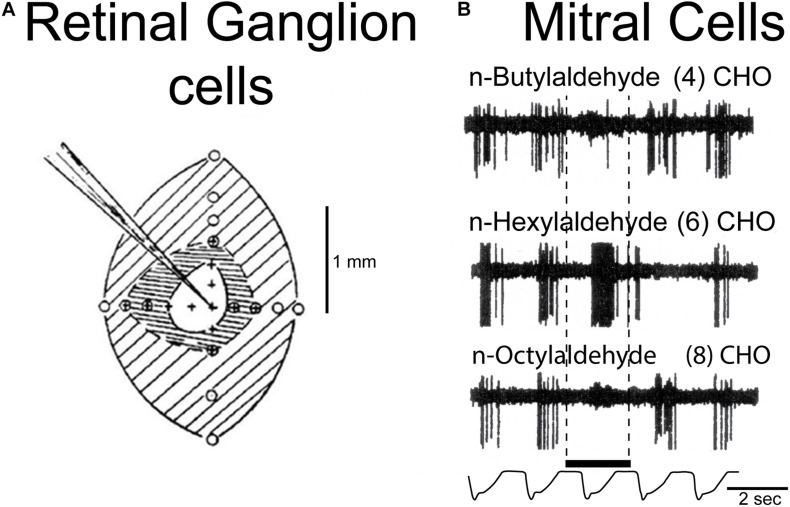
Example of a microcircuit function: lateral inhibition in the retina and olfactory bulb. **(A)** Lateral inhibition in the retina; central excitation of a retinal ganglion cell is surrounded by inhibition, a classical example of spatial contrast enhancement, a fundamental operation in processing spatial patterns in sensory systems. From Kuffler (1953). Center-surround inhibition also underlies color contrast mechanisms in the retina (see text). **(B)** Example of contrast enhancement by “lateral” inhibition in the olfactory bulb, in a chemical series of aldehydes of differing carbon lengths, which heightens contrast between odor molecules by excitation of a mitral cell by one odor molecule type (n-hexylaldehyde 6CHO) and inhibiting responses to neighboring related odor molecules (4)CHO and (8)CHO in the series. Based on Yokoi et al. (1995).

The first and perhaps most studied circuit identified in the olfactory pathway is the dendrodendritic synaptic interaction between mitral and granule cells producing self- and lateral inhibition of mitral cells (Rall and Shepherd, 1968; Bartel et al., 2015; Shepherd et al., 2020). There is evidence that this inhibition mediates contrast enhancement between stronger and weaker activation of mitral cells by different odor molecules (Yokoi et al., 1995). This functions as a type of lateral inhibition of weaker responses to odor molecules, in analogy with lateral inhibition of weaker responses to light stimulation in the retina (see Figure 4). Since the granule cell is the most numerous cell type in the bulb, estimates by some are 100:1 over the mitral cells, and each granule cell has many dozens of spines, each spine supporting a semi-independent computational unit (Woolf et al., 1991; Bartel et al., 2015), the mitral-granule cell microcircuits amplify many fold the computational power of the mitral and granule cells in processing the high-dimensional odor information. The dendrodendritic inhibition is relatively weak, which is believed to be critical in requiring summing with other inputs in order to carry out fine discriminations (Egger et al., 2005). Plasticity of the dendrodendritic synapses is associated with activity-dependent sensitivity (Greer and Halász, 1987).

### Multiple Pathways for Control of Granule Cells

In addition to the dendrodendritic synapses, the granule cell receives many excitatory synapses along its apical dendrite (Balu et al., 2007). They come from multiple sources, including bulbar sources such as mitral cell axon collaterals; centrifugal modulatory fibers from central brain regions; and feedback from the olfactory cortex (see below). Pressler and Strowbridge (2020) found that mitral cells can elicit excitatory postsynaptic potentials (EPSPs) by axon collateral synapses on granule cell bodies and proximal dendrites. In contrast with dendrodendritic synapses, which are relatively weak and depress with repeated stimulation, these are large amplitude and show little depression with repetitive inputs. It is suggested that these synapses more faithfully follow mitral cell firing, thus enabling the granule cells to provide two contrasting versions of odor-driven activity patterns: one lateral inhibitory microcircuit with persistent large amplitude EPSPs in parallel with a second lateral inhibitory microcircuit with actions more conditional on other inputs. Feedback synapses from the olfactory cortex have similar properties, as described below. The feedback from the cortex, plus input from central fibers, provides for odor and context-dependent modulation of mitral cell activity in behaving rats (Kay and Laurent, 1999).

### Mitral Cell Dendrodendritic Lateral Inhibition: Gamma-Beta Oscillations

In addition to lateral inhibition, the dendrodendritically-mediated inhibition gates mitral cell activation through the apical dendrite during odor stimulation, producing an oscillation that begins as faster gamma and transitions into slower beta (Neville and Haberly, 2003; Lagier et al., 2007; Osinski and Kay, 2016). The gamma is produced mainly within the dendrodendritic microcircuit in the olfactory bulb, but the beta requires excitatory depolarizing feedback onto the granule cell from olfactory cortical pyramidal cells (see below). The inhibition is believed to be mediated by a subset of granule cells that connect only to mitral cells. The transmitters are glutamate and GABA, respectively. This closest possible coupling of lateral inhibition and oscillatory activity by the same microcircuit on multiple spines may be critical to the effectiveness of this stage of multidimensional processing.

### Tufted Cell Dendrodendritic Lateral Inhibition

Middle tufted cells are twice as numerous as mitral cells, yet they remain enigmatic in their function. They are located in a layer just underneath the glomeruli (see Figure 3). They appear to be smaller versions of mitral cells, with an apical dendrite connecting to a glomerulus and several secondary dendrites extending laterally. However, tufted cells are genetically distinct, and it is best to think of them as having their own identity. Lateral inhibition of tufted cells is believed to involve a dendrodendritic mechanism similar to that of mitral cells, involving principally a granule cell subset synaptically connected only with tufted cells (Greer, 1987; Mori, 1987). Being smaller and closer to the glomeruli, they have a higher input resistance and are more excitable, responding to lower odor concentrations (Scott, 1981). Their microcircuits thus may have a larger role in odor processing than is usually granted them. This will become apparent in considering their unique axonal connections within the olfactory bulb (see below) and with their output sites in the anterior piriform cortex and olfactory tubercle. Tufted cells may also mediate the large amplitude non-depressing EPSPs in granule cells similar to the actions in mitral cells discussed above. However, their axon collaterals have been described as being quite different in their ramification patterns from mitral cells (see below).

### Combined Mitral-Tufted Cell Dendrodendritic Inhibition

Some granule cells have synaptic connections to both mitral and granule cells (Mori, 1987), so that through both types of reciprocal dendrodendritic connections they may synchronize the lateral inhibition and oscillatory gating of mitral and tufted cells together.

### Mitral Cell Recurrent Axon Collaterals

Prominent in Cajal’s Golgi stains are long recurrent axon collaterals (h in Figure 1) that arise in the deep axons and recur throughout the external plexiform layer. It was early suggested that they spread excitation to tufted cells or granule cells (Shepherd, 1963a, b). Current work is implicating them in the large amplitude granule cell EPSPs discussed above.

## Internal Plexiform Layer

We proceed through the mitral cell body layer to the inner plexiform layer:

### Tufted Cell Axon Collaterals

According to Cajal, these are concentrated in the inner plexiform layer below the mitral cell bodies, and form the “most dense plexus of fibers in the brain” (Cajal, 1911). Tufted cells are glutamatergic. Their axon collaterals extend across the bulb to target their functionally iso-glomerulus in the opposite side, medial to lateral and lateral to medial. There they may connect to the apical dendrites of granule cells passing through the inner plexiform layer; these granule cells could mediate an intense lateral inhibition on surrounding mitral and/or tufted cells (Zhou and Belluscio, 2008). They are in sharp contrast to the mitral cell recurrent axon collaterals, which are widely distributed within the external plexiform layer (see Figure 1).

## Granule Cell Layer

### Granule Cell Neurogenesis

The integration of granule cells into dendrodendritic and axodendritic microcircuits in the external plexiform layer has been discussed above. In addition to the large numbers of granule cells, robust neurogenesis of granule cells in the subventricular zone results in thousands of new granule cells migrating into the olfactory bulb daily (Whitman and Greer, 2007, 2009; Lledo and Valley, 2016). While the full importance of this for the reorganization or plasticity of local circuits has yet to be established, there are lines of evidence suggesting that granule cell turnover may be important in the stabilization of new synapses and the new odor experiences they represent (Forest et al., 2020). This is one of the few instances of sustained neurogenesis throughout life in the mammalian brain (Ming and Song, 2005). We postulate that granule cell turnover may play a crucial role in maintaining and expanding the multidimensional processing capacity of the olfactory system (Saghatelyan et al., 2005) especially in the face of loss of neurons from nasal infections and trauma; it is likely therefore to contribute to the persistence of olfactory guided behaviors following ablation studies of the olfactory bulb.

### Blanes Cells

A type of short axon interneuron in the granule cell layer was first described by a student of Cajal named T. Blanes. Nothing was known about its functional properties until Pressler and Strowbridge (2017) used paired intracellular recordings guided by two-photon microscopy to show that sensory stimulation elicits persistent spiking in Blanes cells which are GABAergic and inhibit granule cells within the granule cell layer. The authors speculate that by modulating bulb neurons with tonic inhibition this may represent a novel mechanism for encoding short-term olfactory information. Blanes cells have also been implicated in coordinating parallel processing in the mitral and tufted cell pathways (Cavarretta et al., 2018).

## Mitral and Tufted Cell Output

The output from the olfactory bulb is carried by the axons of mitral and tufted cells. Our understanding of the output of the olfactory bulb to the olfactory cortex has been greatly enhanced by recent studies in which the mitral and tufted cells have been differentially labeled, giving a clearer view of how they function as parallel pathways. The traditional view is that the axons are carried in the lateral olfactory tract (LOT), as in Cajal’s original diagram (Figure 1), and the traffic is one way, from bulb to cortex. However, modern work is showing that this is one of the most complex interfaces between regions in the brain, involving the cortex as a content addressable memory, feeding back to the bulb as well as forward to the neocortical level for perception.

**FIGURE 5:**
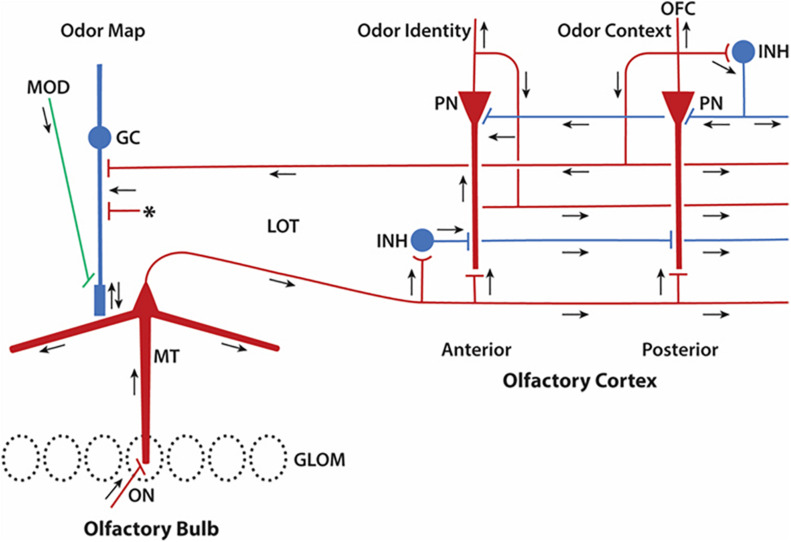
Update of Cajal’s olfactory pathway in Figure 1 based on new knowledge of microcircuits. Close interactions between olfactory bulb and olfactory cortex can be seen to form a multiregional multidimensional processing unit underlying olfactory perception. Red indicates excitatory synaptic action, blue inhibitory synaptic action. Arrows indicate direction of impulse propagation and synaptic action. GLOM, glomeruli; GC, granule cell; INH, inhibitory interneuron; LOT, lateral olfactory tract; MOD, modulatory systems; MT, mitral/tufted cell; PN, pyramidal neuron; OFC, orbitofrontal cortex. Asterisk denotes excitatory inputs to the granule cell from multiple sources (see text). Note that lateral circuits in the olfactory cortex for processing the sensory input from the LOT are in the excitatory and inhibitory layers closest to the LOT, whereas circuits for associative processing are closest to the PN cell bodies. Feedforward inhibition predominates in the APC, inhibitory feedback in the PPC. Based on many authors (see text).

We can attempt only an outline of these complex mechanisms, which lie at the heart of how the olfactory pathway deals with its high dimensional information. We will not be able to cover all the structural aspects of the olfactory cortex, but rather focus on what we will term the core multiregional multidimensional processing unit formed by the interactions within and between the olfactory bulb and olfactory cortex, as depicted in Figure 5. This diagram is in a format similar to that of Cajal’s diagram in Figure 1 to facilitate appreciating the advances that have been made in modern studies.

## Piriform Cortex Contains Different Areas

Current research is revealing anatomical and functional subdivisions within the piriform cortex. Several discrete pulses in evolution of piriform cortex size can be traced in the fossil record (below), and this evidence is consistent with its division into anatomically distinct anterior and posterior parts. The anterior olfactory nucleus (now considered a part of the anterior piriform cortex) is also involved (Brunjes et al., 2005; Levinson et al., 2020). Here we will focus on the olfactory cortex, the part of piriform cortex receiving direct input from the mitral and tufted cells of the olfactory bulb.

### Olfactory Cortex Activation

The olfactory cortex is traditionally considered a three-layer cortex, with a superficial layer where the input fibers of the lateral olfactory tract (LOT) interact with the distal dendrites of the pyramidal cells; a layer of pyramidal cell bodies; and a deepest layer of interneurons and fibers. Mitral and tufted cell axons course in the lateral olfactory tract giving off collaterals to the apical dendritic tufts of pyramidal cells.

This means that a wave of excitation passes through the cortex, but this wave may not have functional significance for the individual pyramidal cell. The propagation velocity of an action potential in the 1 μm diameter axons is approximately 1 mm/sec, so although propagation in the LOT produces a wave of excitation, if pyramidal cells are 50 μm apart there is only a 0.05 ms (50 microsecond) difference between them, meaning near-synchronous activation of neighboring pyramidal neurons. Neighboring pyramidal cells may therefore function as if they are in a cluster or column together, whereas more separated cells act more as if they are activated in sequence. The near simultaneous activation of neighboring olfactory cortical pyramidal cells enabled a current source density analysis to reveal the excitatory lateral association fibers in the cortex (Haberly and Shepherd, 1973).

As noted, the cortex has two main areas, anterior and posterior. The pyramidal cells give off collaterals that form association fibers that lie in several layers. In the anterior area the fibers are directed from anterior to posterior in a superficial layer near the LOT, whereas connections made by pyramidal cells in posterior cortex are in a deeper layer oriented from posterior to anterior (Figure 5). These fibers and their connections extend widely throughout the cortex and are remindful of the extensive associational axonal network characteristic of the face area in the neocortex (Haberly, 1985). Through this network pyramidal cells act as an auto-associative network which functions as a content addressable memory. This is a high level function equivalent to memory mechanisms in other cortical areas such as hippocampus, and in artificial network devices (Gire et al., 2013).

### Olfactory Cortex Oscillatory Activity

Odor stimulation also activates oscillatory activity, first gamma (35–90 Hz) and then beta (15–35 Hz) (Neville and Haberly, 2003), which via the mitral cell axons to the olfactory cortex and the pyramidal cell association fibers to the olfactory bulb are tightly coupled to the same waves in the olfactory bulb (see step 14). Gamma appears to be associated with the exploratory phase of odor stimulation, whereas beta is associated with the reward of an odor (Wilson and Barkai, 2018). A computational model has supported the electrophysiological evidence that the dendrodendritic microcircuit in the olfactory bulb is involved in generating both of these frequencies, depending on the balance between the smaller excitatory depolarization of the mitral cell odor response and the larger depolarization due to cortical feedback (Osinski and Kay, 2016).

### Posterior Cortical Area

This is the main cortical region for olfactory processing before the neocortex (Wilson and Stevenson, 2006; Bekkers and Suzuki, 2013; Gire et al., 2013; Fournier et al., 2015), and the region of piriform cortex that undergoes the greatest degree of evolutionary expansion (Rowe et al., 2011). Electrophysiological evidence by Haberly and Bower (1984, 1989), and Haberly (1985) showed that the pyramidal cells are characterized by axons whose association collaterals branch widely throughout the cortex. These and subsequent studies have changed views of the olfactory cortex, from a simple relay from the olfactory bulb to the neocortex, to a high level association cortex analogous to the visual face area. This view required the olfactory bulb to function in analogy with a primary or secondary neocortical area in providing the direct input to the olfactory cortex acting as a higher olfactory association cortex, even though it is only a three-layer, not six-layer, cortex.

Pyramidal neurons and their interneurons form the feedforward and feedback circuits characteristic of cortical processing. Evidence for the functions of these circuits in olfactory cortex is beginning to emerge. As summarized by Bekkers and Suzuki (2013), the input from the olfactory bulb is an “odotopic image” resulting from filtering the incoming sensory data, normalizing it through a balance of excitatory and inhibitory circuits, carrying out feature extraction by inhibitory actions, and decorrelating overlapping activity patterns through lateral inhibition to enhance pattern separation. This is projected onto the piriform cortex whose main function is to convert these “images” of odors, spatial activity patterns distributed across olfactory glomeruli, into unified internal “odor objects” as distributed across interconnected piriform pyramidal cells (Wilson and Sullivan, 2011). These internal objects are the end results of the multiple steps of hyperdimensional processing, which are further projected from the posterior piriform cortex to the orbitofrontal cortex as the basis for odor perception and to other regions for odor related behavior (see below).

Together these olfactory cortical microcircuits carry out a sequence of two basic types of operations (Bekkers and Suzuki, 2013). In the anterior piriform (olfactory) cortex, afferent inputs from the olfactory bulb dominate (Figure 5), making it adapted for encoding the “identity” of odor molecules. In the posterior (olfactory) piriform cortex, association fibers dominate, making it adapted for matching the sensory identity with the stored identity, creating the “quality” or behavioral significance of the odor for the perceiving individual. This operation gives the piriform cortex the property of a “content addressable memory” mentioned above, an essential function found across nearly all mammals, including humans (Gottfried, 2010).

### The Core Olfactory Multiregional Multidimensional Processing System

Until now we have emphasized the progression of processing from the periphery into the brain. However, a key to the basis for processing the high dimension olfactory information is feedback, so that a processing unit takes in not only the sensory input but the sensory input that is continually combined with feedback from the processing unit itself. As expressed by Pressler and Strowbridge (2017), “Working together, local bulbar and cortical feedback excitatory pathways may function to generate sparse but highly odor-specific GC [granule cell] discharges that facilitate olfactory discrimination …”. A final contribution to the integration is from the previous integrated inputs stored in memory.

We can summarize this new view of Cajal’s diagram in relation to Figure 5. Sensory input is processed in the olfactory bulb to form the output to the olfactory cortex. The olfactory cortex processes this information in the sequence of identification and contextual significance as described previously. Lamination of the cortex provides for stages of processing from sensory to central and stores the central result as a content addressable memory. This is fed back from the cortex to the granule cells in the olfactory bulb to be merged with the incoming information from the olfactory bulb, and the operations update on a continual basis. The feedforward and feedback targets are at different sites on the granule cell, enhancing the different types of information that are combined there. One can say that this fiendishly ingenious organization of neuronal microcircuits compresses a maximum of continuous information formatting and reformatting in a minimum of space. In this way, information in the olfactory pathway is subjected to constant reevaluation and updating for perceptual creation and behavioral relevance. The limited space in fact works to enhance the operations by requiring their close interactions.

### Output From the Posterior Olfactory Cortex

**FIGURE 6:**
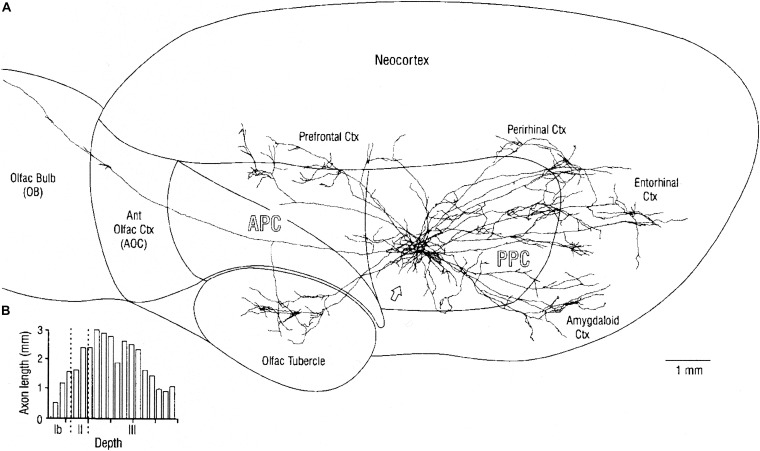
**(A)** Overview of mitral cell axon projecting to the posterior piriform cortex (PPC), and reconstructed axonal arbors of two neighboring layer II pyramidal cells. Stained by *in vivo* intracellular injection in the rat. Note that association axons extend through most of the piriform cortex as well as to distant regions with different output functions. **(B)** Plots of depth of association fibers of 5 identified layer II pyramidal cells. Note that fibers from posterior piriform cortex are densest in layer III among pyramidal cell bodies and basal dendrites and inhibitory interneurons, whereas association fibers from anterior piriform cortex connect to apical dendrites near the sensory input from the LOT. AON, anterior olfactory nucleus; APC, anterior piriform cortex; PPC, posterior piriform cortex; and olfactory tubercle are three-layer cortex, Neocortex is six-layer cortex. See text. From Haberly (2001).

The pathway for olfactory perception continues from the olfactory cortex to the primary olfactory receiving area in the orbitofrontal cortex (Chen et al., 2014; Diodato et al., 2016) (Prefrontal cortex in Figure 6). Some olfactory cortical pyramidal cells connect directly to the orbitofrontal region, which is the level at which odor perception is believed to occur; others project first to the endopeduncular nucleus, and others to the mediodorsal thalamus, with further projection to the orbitofrontal cortex. These different routes to the neocortex might be related to different kinds of olfactory information issuing from the olfactory cortex.

An important aspect of the output from the posterior piriform olfactory cortical area is that multiple connections are made by this association area to external regions not exclusively olfactory. Figure 6 shows a typical example, in which labeled cells are shown to project not only to the prefrontal cortical area (which in the rat includes the orbitofrontal area and the insula) but also to the olfactory tubercle (a ventral extension of the striatum), amygdala, entorhinal area, and perirhinal area (with further connection to the hippocampus). These connections provide ways which bring the olfactory input into close involvement with areas generating learning, emotion and olfactory-guided behavior. Of special interest is evidence showing direct connections of the tufted cells to the olfactory tubercle (Nagayama et al., 2010), and the role of the tubercle in olfactory-guided reward behavior (Gadziola et al., 2020). Given these multiple types of output, much of the olfactory cortex must reflect an organization of the olfactory input that can be sent to these distinct roles in these differing output systems. It may be that this function of output to different targets is compressed within the olfactory cortex but more distributed within different cortical regions for example in the visual system.

## Modulation by Centrifugal Fibers

In addition to these specific processing mechanisms, nervous systems provide for mechanisms for their modulation dependent on behavioral states. The olfactory microcircuits are heavily modulated by central systems.

### Cholinergic

Cholinergic fibers to the olfactory bulb arise within the horizontal nucleus of the diagonal band. Cholinergic receptors enforce the responses in mitral, tufted and PG cells, and prolong the responses of granule cells, enhancing perceptual discrimination (Chaudhury et al., 2009) (see MOD, Figure 5).

### Noradrenaline (NA)

These fibers arise in the locus coeruleus and innervate the olfactory bulb and the olfactory cortex. The released NA suppresses spontaneous activity but does not affect sensory responses. It also modulates odor habituation and discrimination (Guerin et al., 2008) and the stability of odor memory (Linster et al., 2020).

### Serotonin (5HT)

5HT fibers from the dorsal raphe densely innervate the olfactory glomeruli. Direct application of 5HT to a glomerulus modulates glomerular network activity, increasing external tufted cell excitation of mitral cells, short axon cells and periglomerular cells; inhibiting glomerular interneurons; triggering action-potential-independent GABA release from short-axon cells; and increasing spontaneous mitral cell firing without enhancing responses to sensory input (the opposite of the effect of NA). It is suggested that this can raise mitral cell sensitivity while maintaining dynamic range (Brill et al., 2016).

In summary, microcircuit functions are continuously modulated by central systems in the brain in relation to different behavioral states.

## Evolution of Olfactory System Microcircuits

A discussion of olfactory microcircuits is not complete without taking advantage of evidence that can be gained from the unique evolutionary path followed by the olfactory system. For this purpose, we continue to pursue a novel conceptual approach in which paleontological studies give insight into olfactory sensory and brain development (Rowe et al., 2011), and modern anatomical and physiological studies, through evidence regarding microcircuits, provide insight into the functional significance of the paleontological record (Rowe and Shepherd, 2016; Shepherd and Rowe, 2017; Rowe, 2020f).

We postulate that the multiplicity of microcircuits for sensory processing in the olfactory pathway reflects the unique evolution of the olfactory pathway compared with vision and other sensory systems. The importance of vision in human life has meant that the visual pathway is the most studied of the sensory systems, and the olfactory pathway among the least studied, but from the perspective of evolutionary origins the significance of the two systems is surprisingly, to a great extent, the opposite. We consider first the paleontological record, followed by evidence regarding the evolution of the cortical microcircuits.

**FIGURE 7:**
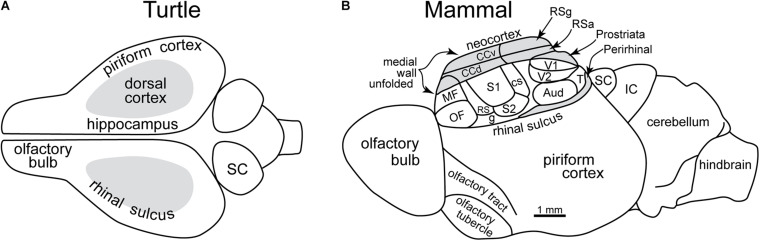
Early evolution of the forebrain dominated by the olfactory cortex. **(A)** Dorsal view of the forebrain of a reptile (turtle), showing the three main areas: olfactory, hippocampal, and dorsal (visual). **(B)** Lateral view of reconstructed mammalian “ancestral forebrain cortex”: OF, orbitofrontal area; MF, medial frontal area; S1, S2, RS, CS: primary, secondary, dorsal and caudal somatosensory areas; g, gustatory area; V1, V2, T: primary, secondary and temporal visual areas; Aud, auditory areas; CCv, CCd; ventral and dorsal cingulate areas; RSg, RSa, retrosplenial granular area and agranular areas; SC, superior colliculus; IC, inferior colliculus. **(A)** Based on Shepherd (2011). **(B)** Based on Molnar et al. (2014).

As we have seen, olfactory processing has a privileged position in the brain, beginning in the olfactory bulb, a structure derived from the highest level of information processing, the forebrain. Across vertebrates, the forebrain cortex has a fundamental organization. It is divided into three areas: hippocampal on the medial side, olfactory on the lateral side, and a dorsal cortex between that relates (primitively) to the visual system (Figure 7A). However, most of the input from the retina is routed to the optic tectum (superior colliculi in mammals) for rapid visual reflexes and motor responses. All of these cortical areas in non-mammalian vertebrates (with the possible exception of late stem-mammals) have a three-layer construction, consisting of a single layer of pyramidal cell bodies with an underlying layer of axons and an overlying layer of the dendrites of the pyramidal cells and of interneurons (see below). This basic architecture reflects ∼500 million years of conserved organization in most vertebrate clades. It also reflects the ancestral condition that was transformed in mammals as their uniquely elaborated olfactory system and neocortex evolved.

Evidence that olfaction was an important driving factor in nearly all of the major clades across the entire 500 million year history of vertebrate evolution lies in the fact that OR genes are the largest multigene family in vertebrates (Niimura, 2009). The OR genome has been found to evolve at faster rates than other systems, and this is especially true in the 400 million years of tetrapod evolution, as vertebrates adapted to terrestrial environments (Yohe et al., 2020). Just as the tetrapod visual system evolved in response to the far greater diversity of reflective objects on land than in the water (Walls, 1942), the tetrapod OR genome evolved more rapidly than other protein coding genes (Yohe et al., 2020) as it accommodated a more diverse and more rapidly changing chemical environment encountered in terrestrial ecosystems.

In mammalian evolution, elaboration of the olfactory system exceeded all other tetrapods, an extreme example of what has been called “evolutionary overdrive” (Yohe et al., 2020). This can be measured by the relative size of the mammalian olfactory genome, the unique complexity of olfactory microcircuitry described above, at anatomical levels in the size and complexity of neural and skeletal structures that are induced in ontogeny as OR genes are expressed, and its morphogenic impact on skull design (Rowe, 1996a, b). Whereas ‘fish’ (non-tetrapod vertebrates) have only ∼100 functional OR genes, an estimated ∼1,200 functional OR genes were present in the ancestral mammal, as inferred from living species (Niimura and Nei, 2006; Niimura, 2012; Niimura et al., 2014). This increase was probably the product of multiple tandem gene duplications such that the OR genes comprise the largest gene family in the mammalian genome and ∼5% of all protein coding genes (Nei et al., 2008; Yohe et al., 2020). In a study of human OR genes, Mainland et al. (2014) found that function-altering polymorphisms in genes for OR subtypes differ on average in 30% of those genes when comparing two individuals. These transformations occurred over a span of ∼150 million years, between the divergence of the mammalian total clade from other amniotes, and the origin of crown Mammalia ∼170 million years ago (Rowe, 2020f).

Combining the evidence from genes, paleontology and microcircuits, the basic evolutionary insight these small creatures with only general terrestrial adaptations depended for survival on developing a powerful sense of smell as they rooted around searching for food in the dense and sometimes toxic or infectious underbrush. Molecular research has shown the extremely large gene family of ORs and OSNs involved. Paleontological research shows the large nasal surfaces coupled with large olfactory bulbs and olfactory cortical areas for processing this input. And microcircuit research on extant organisms reveals corresponding powerful processing mechanisms within those large structures. Finally, the system has redundancy built in at each processing stage so that it is resistant to being damaged by potentially toxic and infectious environments. We summarize the basic steps that occurred.

### Early Pan-Mammals

The beginnings of the mammalian olfactory system can be traced back to the earliest members of *Pan-Mammalia* as it diverged onto its own evolutionary trajectory between 340 – 322 million years ago (mya) (Didier and Laurin, 2020). At this early stage, the braincase was only partly enclosed by bone, and the obtainable evidence points to a primitive narrow tubular forebrain with three-layer dorsal cortex, and small olfactory bulbs appressed against the rostral telencephalon (Rowe, 2020f). Possibly a modestly expanded repertoire of OR genes distinguished Pan-Mammalia from near the start of its history (Rowe and Shepherd, 2016; Rowe, 2020e,f,g; Rowe et al., 2005). Nevertheless, this early history was dominated by improvements in terrestrial locomotion and agility, and vision probably greatly overshadowed olfaction in importance (Rowe, 2020f).

### Cynodontia

The balance between vision and olfaction began to shift ∼250 mya, with the origin of Cynodontia, a clade that includes crown mammals and their closest extinct relatives (Rowe, 2020a,d,f). Wholesale reorganization of the skull and skeleton suggests that olfactory evolution had moved into “overdrive” and the expression of duplicated OR genes was probably underway (Rowe and Shepherd, 2016). Details of the skeletal modifications are described and illustrated elsewhere (Rowe and Shepherd, 2016; Rowe, 2020c, f). What is relevant here is that the first cynodonts mark a first major pulse in encephalization, specifically in enlarged olfactory bulbs, implying an increase in numbers of glomeruli, and expansion of the posterior piriform cortex. These were associated with the origins of an occlusal dentition and secondary palate which provide for the new behaviors of food mastication and retronasal olfaction, and a far more rapid rate of evolution in the dentition that signaled rapid dietary diversification. This corresponded to a reduction in body size on the direct line leading to mammals; early cynodonts were about the size of a domestic cat (Rowe and Shepherd, 2016; Rowe, 2020c, f).

There is no evidence suggesting that six-layer neocortex had yet appeared, and endocasts of these early cynodonts resemble those of the earliest stem-amniotes more closely than they resemble endocasts of living mammals. Nevertheless, OR gene duplications seem very likely in these extinct relatives of living mammals, promoting fixation of novel functional mutations (Niimura, 2012), and a high rate of codon substitution relative to nucleotide substitution reported in living mammals had probably begun to promote diversification of novel odorant binding proteins (Yohe et al., 2020), initiating an immense expansion in the dimensionality of perceptible odorants that most living mammals enjoy. Thus, OR gene duplications that occurred leading up to the origin of the crown clade shifted mammalian OR gene evolution into what Wagner (2014) refers to as a “novel variational modality” characteristic of other anatomical systems of repeated structures, including the cynodont dentition (Rowe, 2020f).

### Mammaliamorpha

The next important event coincided approximately with the origin of Mammaliamorpha (Rowe, 2020c, d), roughly 230 mya. Endocasts show a greater degree of expansion of the brain overall, with a larger olfactory bulb separated from the dorsal cortex by a shallow constriction, and the interhemispheric sulcus is marked in the endocast (Rodrigues et al., 2014; Wallace et al., 2019; Kerber et al., 2021). μCT studies reveal that the nasal capsule adjacent to the olfactory bulbs had begun to ossify. Small bony elements further forward in the nose may be primordial ossified turbinals, indicating elaboration of the olfactory epithelium (Kielan-Jaworowska et al., 2004; Rodrigues et al., 2014; Wallace et al., 2019). The piriform cortex also expanded to the degree that its posterior pole was as wide as the cerebellum. Evidence of increased high frequency audition is also present, but the overall impression is elaboration of each of the ontogenetically interdependent areas of the olfactory system.

### Mammaliaformes

The next major pulse in pan-mammal encephalization came with the origin of *Mammaliaformes* ∼210 mya (Rowe, 1988, 2020c, 2020d, 2020f). Early mammaliaformes were all miniaturized, and for the next ∼140 million years, few members of this clade exceeded the size of a shrew or small rodent (this possibly occurred in the ancestral mammaliamorph). μCT enabled visualization of a digital endocast of the basal mammaliaforms *Morganucodon* and *Hadrocodium* (Rowe et al., 2011) which showed olfactory bulbs that are quite large and inflated, and separated from the inflated forebrain by a deep circular fissure. The piriform cortex, especially its posterior component, had expanded laterally and taken on its namesake ‘pear-shape.’ The endocasts in these fossils now closely resemble those of crown mammals, which appeared ∼40 million years later, and their encephalization quotient nearly reached the level of the least-encephalized crown mammals (Rowe et al., 2011). Nerves from hair follicles and their attendant musculature provided a flood of new peripheral input to the brain (Rowe et al., 2011; Rowe, 2020f). Enhanced high frequency hearing is also implicated in driving encephalization. However, the principal driver, unquestionably underway since the earliest cynodonts, once again was olfaction.

### Mammalia

With the origin of Mammalia ∼170 million years ago, neocortex was unquestionably present, and the olfactory system consisted of up to ∼1,200 OR gene types or more whose expression induced the ontogenetic cascade described above. Comparisons with living mammals enable the reconstruction of brain organization in the ancestral mammal as shown in Figure 7B (Molnar et al., 2014; Rowe and Shepherd, 2016). The large olfactory cortex reflects the large processing load of the massive OSN input and the dominance of smell in driving the early evolution of the forebrain. This increased processing and increased areas presumably extended to olfactory prefrontal and orbitofrontal neocortex areas. By the same token, the enlargement of the areas for other senses reflected the roles that vision, audition, touch and taste began to play in the mammalian adaptations for survival and spread. An interesting possibility is that the initial drive toward the neocortex included the increased processing load from olfactory cortex as well as from the other sensory, motor and central systems.

**FIGURE 8:**
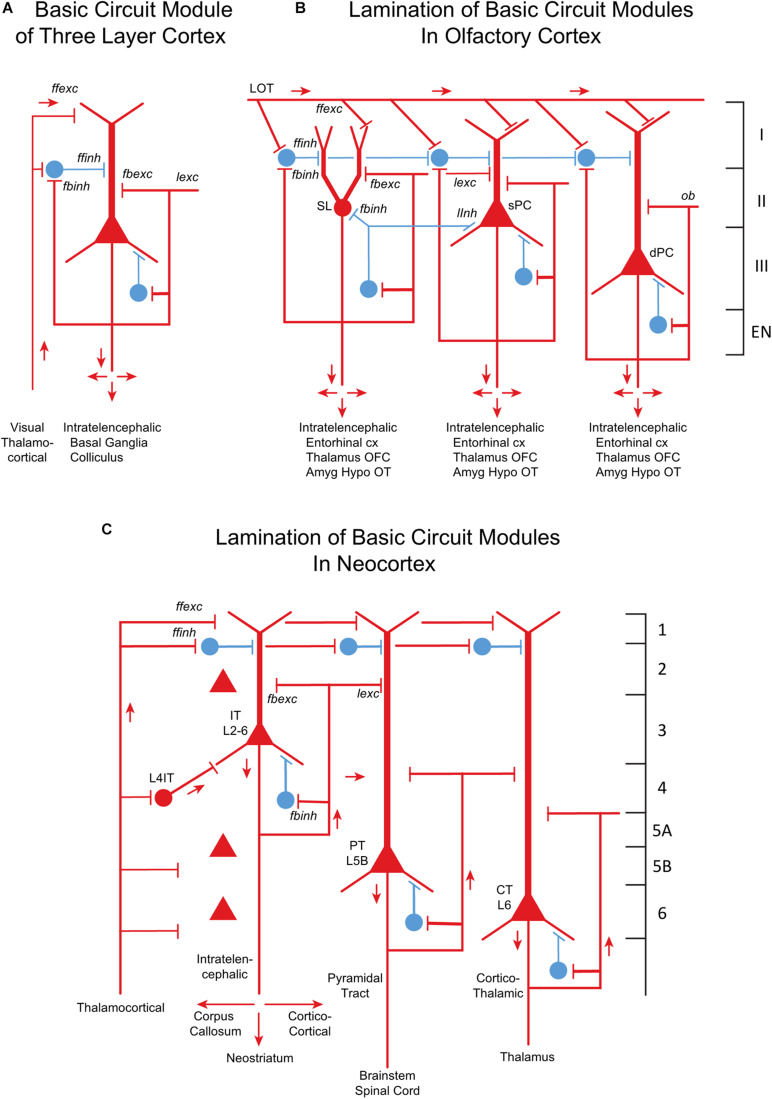
From three-layer to six-layer cortical microcircuits. **(A)** Simplified basic cortical module of ancestral three-layer olfactory cortex, hippocampus and dorsal cortex. This is the basic cell organization of these three areas shown in Figure 7. Based on Kriegstein and Connors (1986) and Shepherd (2011). PC, pyramidal cell. Abbreviations of functional actions: ffexc, feedforward excitation; ffinh, feedforward inhibition; fbexc, feedback excitation; fbinh, feedback inhibition; lexc, lateral excitation; linh, lateral inhibition. **(B)** Olfactory cortex: lamination of basic circuit modules. **(C)** Mammalian neocortex: lamination of basic circuit modules. Abbreviations as in **(A)**. Laminae for the cell types are indicated. Presumed excitatory cells shown in red, inhibitory cells shown in blue. Based on Shepherd (1988) and Shepherd and Rowe (2017). Martin-Lopez et al. (2019) have shown how the piriform laminae follow a selective developmental and migratory program established by cell lineage.

Cortical microcircuits obviously played an essential role in that survival. Based on a postulate of Kriegstein and Connors (1986), a possible progression from the earliest forebrain cortex through olfactory cortex to neocortex is summarized in Figures 8A–C. The diagrams illustrate the underlying principle of a “canonical cortical integrative unit,” exhibited by reptilian cortex (Figure 8A), of a principal neuron, the excitatory pyramidal cell, activated by feedforward excitation (ffexc) and modulated by recurrent axon collaterals that mediate feedback excitation (fbexc) and lateral excitation (lexc). This excitatory input is balanced by an interneuron (or several interneurons) that mediates feedforward inhibition (ffinh), and axon collaterals that mediate feedback (fbinh) and lateral (linh) inhibition. In olfactory cortex (Figure 8B), pyramidal cells are differentiated into layers containing semilunar cells (SL), superficial pyramidal cells (sPC) and deep pyramidal cells (cPC). Each type appears to be associated with excitatory and inhibitory connections elaborated from the three-layer circuit model. The cell differentiation, layering, and distribution into different areas reaches its extreme in neocortex (Figure 8C), but the diagram enables the basic canonical cortical integrative unit to be recognized.

Studies of piriform cortex, like those of neocortex, using gene expression methods and labeled neuron physiology are finding that the cells in the two cortices share many genetic markers (summarized in Diodato et al., 2016; Klingler, 2017). In both there is an inside-out sequence of generation of pyramidal cells, interneurons, and glia, except for layer II in olfactory cortex (Martin-Lopez et al., 2019). During development, excitatory pyramidal cells come from the lateral migratory stream (Bai et al., 2008), interneurons (in rodents) from the ganglionic eminence (Marin and Rubenstein, 2003). During development the pyramidal neurons and interneurons come together to form the feedforward and feedback microcircuits that characterize the canonical integrative units mediating cortical processing (Figures 8A–C).

In summary, with a humble beginning in early pan-mammals, significant elaboration occurred in early Cynodontia and early Mammaliamorpha, accelerating considerably in the miniaturized early Mammaliaformes, and finally in crown Mammalia. The olfactory cortical microcircuits elaborated more complex layers with internal feedback and lateral excitatory microcircuits, multiple areas, feedback to the olfactory bulb, and multiple interconnections with output sites, as seen in modern opossums and shown in the diagrams of Figures 6, 7B, 8. The number of OR genes is reflected in the numbers of olfactory glomeruli, meaning an increase in the glomerular microcircuits processing the increase in numbers of receptors and receptor types. Our current picture of OR evolution is consistent with the hypothesis that peripheral sensory arrays influenced central organization, and that through epigenetic population matching cortical reorganization and relative increases in brain size may have been driven to a significant degree by connectional invasions from peripheral cell populations and sensory structures such as teeth and hair, and especially OSNs (Katz and Lasek, 1978; Krubitzer and Kaas, 2005; Streidter, 2005; Rowe and Shepherd, 2016; Rowe, 2020f).

## Discussion

How does the evolution of an increased area of six-layer microcircuits relate to our interest in high dimension processing in olfaction and smell? Mammalian vision is associated with six-layer neocortex (including additional sublayers in V1); why did olfaction stay with its simple three-layer cortex (although in echidnas, at least, a four-layer piriform cortex did evolve)? From our review of olfactory microcircuits, it appears that the multiple overlapping microcircuits contained within the pathway enable the system to achieve sufficient high dimension processing ending in the three-layer olfactory cortex where it begins already to connect to multiple sites for behavioral output (Figure 6). As we saw, olfactory processing, beginning with agonist and antagonist pharmacological interactions at the initial step of sensory transduction in the olfactory cilia, passes through over 20 lateral processing circuits up to the olfactory cortex. Induction by the olfactory epithelium of a complex internal skeleton is an associated phenomenon.

Given their distribution across vertebrates, the basic mechanisms for processing the high dimension stimuli of odor molecules were present at the outset of vertebrate evolution, which occurred in an aquatic medium in which olfaction has a lower dimensionality. They persisted in the expanded three-layer cortex of the first tetrapods, where OR evolution increased its rate in terrestrial environments that have the highest olfactory dimensionality. And in the evolution of the total mammalian clade, olfactory mechanisms achieved the unique measure of complexity described above. The olfactory system has thus always had a strong representation at the cortical level even with only a three-layer cortex.

One says “only three-layer cortex,” assuming that the six-layer neocortex is necessary to achieve the higher cognitive functions exemplified by the visual system. However, recent behavioral experiments have revealed that higher visual perceptual tasks such as human facial recognition are achieved by the three-layer cortex of fish, more primitive even than the organization shown in Figure 8A. Archer fish, which spit at their targets above the water, were trained to distinguish one among 44 human faces differing only subtly in features, spitting at a conditioned stimulus face on a monitor above the water surface (Newport et al., 2016). The study showed that this human higher cognitive function can be mediated by a vertebrate lacking a neocortex and an evolutionary history lacking adaptation to this function. Related studies have shown that facial discrimination can also be made by bees and birds which also lack a neocortex; birds can also categorize faces based on expressions and gender (reviewed in Newport et al., 2016).

These results show that animals without a neocortex can nonetheless carry out fine detail pattern discrimination often within only a single session. The implication for the olfactory bulb and three-layer olfactory cortex is clear. There is much evidence for the formation of finely detailed “odor images” (see “odotopic” maps above) in the olfactory bulb (Shepherd, 2010) and widely distributed “odor objects” in the olfactory cortex (Wilson and Barkai, 2018). Three-layer cortex can be hypothesized to be well able to carry out detailed pattern discrimination of these images and objects. It then passes on to neocortex the responsibility for higher cognitive functions that have enabled the elaboration of mammalian and especially human behavior.

## Future Microcircuit Approaches

We have begun to identify the sequence of microcircuits and their functions that constitute the neural basis of odor perception. We encourage similar efforts in other sensory pathways to build a unified understanding of the neural basis of sensory perception with its evolutionary history.

Applied more generally, this could provide an enhancement of the approach used in *C. elegans*, identifying all synapses in a simple nervous system, to identifying the main sequence of microcircuits in different specific mammalian systems, giving a functional meaning to the organization of the synapses and an evolutionary significance to the microcircuit actions underlying mammalian behavior.

## Data Availability Statement

Publicly available datasets were analyzed in this study. This data can be found here: University of Texas High-Resolution X-ray Computed Tomography Facility (http://www.ctlab.geo.utexas.edu), and on Digital Morphology an NSF Sponsored Digital Library at the University of Texas (http://digimorph.org/specimens/Monodelphis_domestica/adult/ and http://digimorph.org/specimens/Zaglossus_bartoni/).

## Author Contributions

All authors listed have made a substantial, direct and intellectual contribution to the work, and approved it for publication.

## Conflict of Interest

The authors declare that the research was conducted in the absence of any commercial or financial relationships that could be construed as a potential conflict of interest.
